# Chinese Virtues and Resilience among Students in Hong Kong

**DOI:** 10.3390/ijerph20043769

**Published:** 2023-02-20

**Authors:** Xiaoxue Kuang, John Chi-Kin Lee, Junjun Chen

**Affiliations:** 1 Department of Education, School of Education (Normal School), Dongguan University of Technology, Dongguan 523808, China; 2 Department of Curriculum and Instruction, The Education University of Hong Kong, Hong Kong SAR, China; 3 Department of Education Policy and Leadership, The Education University of Hong Kong, Hong Kong SAR, China

**Keywords:** Chinese virtues, resilience, quantitative, gender, school grade level

## Abstract

Character strengths and training have a great impact on students’ whole-person development. This study examined the applicability of the Chinese virtues questionnaire (CVQ) and the relationships between students’ perceptions of virtues and resilience in Hong Kong, SAR, China. A total of 2468 pupils from primary and secondary schools in Hong Kong were recruited as the sample for this study. The results of confirmatory factor analysis (CFA) supported a measurement model of Chinese virtues, and the results of structural equation modeling (SEM) suggested that Chinese virtues were positively related to positive resilience and succumbing. Significant relationships were found between gender and students’ positive resilience, and school grade level showed a significant impact on the Chinese virtues, which in turn affect resilience. Student resilience could be enhanced by nurturing virtues and related character strengths, keeping in mind the role of gender and grade level.

## 1. Introduction

Character strengths are positive traits that appear in people’s thoughts, emotions, and behaviors [1]. As such, character development has a central role in the development of individual students and society as a whole [2]. Peterson and Seligman [1] categorized 24 character strengths into six virtues (e.g., courage, humanity, justice, temperance, transcendence, and wisdom), and these have been widely applied in educational contexts. These six virtues can be found in many cultures, including Buddhism in South Asia, Athenian philosophy and Christianity in the West, and Confucianism and Taoism in China [3]. Shek and Ma [4] found that nearly 70% of teacher respondents perceived a decline in morality among Hong Kong adolescents. More than half of respondents perceived their own students as friendly (84%), kind (80%), and empathetic (64%), but over 70% thought that their students still needed to improve in terms of sense of responsibility (73%), emotional competence (98%), and ability to cope with adversity (99%).

Despite the need to boost virtues among students, the predictors and outcomes of character strengths and virtues are relatively underexplored. Niemiec [5] revealed, both theoretically and empirically, six major functions of character strengths, a vital one of which was nurturing resilience. Resilience is also an important attribute in education and personal development [6]; to enable positive development and psychological resilience in children and adolescents, it is essential to understand the underlying concepts [7]. In line with these findings, demographic factors, such as gender or school grade level, influence how protective factors (i.e., virtue or character strength) are associated with resilience [8]. Some studies have shown that gender and age are important predictors of resilience [9,10,11], while the impact of gender is complex for different groups of children [9]. Most studies have focused on the adult group aged 18 or above [10,11]; few study has considered the potential effect of the different grades in primary or secondary school when youth develop their key personality.

Despite the acknowledgment that character strengths and virtues are important, current studies remain limited. First, based on functional equivalence and conceptual equivalence, Duan et al. [12] suggested that some items of the international measurement of virtue, values in action inventory of strengths (VIA-IS), might not be applicable in the Chinese context. Second, although subsequent studies revealed that character strengths relate positively to positive outcomes, including life satisfaction [13], well-being [14], and adaptability [15] in different contexts, there is a lack of scholarship on student virtues and resilience in Hong Kong. Third, despite the growing literature on student virtues, the potential impact of individual factors (i.e., both gender and school level) on virtue and resilience are not well understood. To fill in the research gaps and address the concerns mentioned above, this study was set in Hong Kong, an international Asian city, where there are influences from and interactions between Chinese cultural heritage and Western cultures. There is an increasing emphasis on life and values education [16,17,18] as well as Chinese cultural education, as revealed by the issuance of the values education curriculum framework by the Education Bureau [19]. There is also mention of the importance of enhancing student resilience [20]. This study examined the applicability of the Chinese Virtues Questionnaire (CVQ-96) for primary and secondary students in Hong Kong and the possible relationships between student perceptions of virtues and resilience in the Chinese context, as well as the role of gender and school grade level in this relation.

## 2. Literature Review

### 2.1. Character Strengths and Virtues

The inter-relationship between virtues and character strengths has been documented in the literature [1]. Character strengths are personality characteristics reflected in a person’s thoughts, emotions, and behaviors [1]. These can be defined as positively valued and moral traits, which develop an individual’s growth, flourishing, and moral excellence [21]. In addition, the concept of virtue, according to Aristotle, is related to moral characteristics, such as honesty, temperance, courage, justice, and liberality [22] (p. 172). Some of these virtues have been employed by psychologists as elements or components of character strengths [23]. Peterson and Seligman [1] identified six virtues in 24 character strengths, including courage (e.g., bravery, industry, integrity, zest); humanity (e.g., kindness, love, social intelligence); justice (e.g., teamwork, fairness, leadership); temperance (e.g., forgiveness, modesty, prudence, self-control); transcendence (e.g., appreciation of beauty, gratitude, hope, humor, spirituality); and wisdom (e.g., creativity, curiosity, judgment, love of learning, perspective). This study asserts that these six virtues have been relevant for at least three centuries in a wide variety of cultural, religious, and traditional settings [1].

### 2.2. Measurement of Virtues

It was anticipated by the Peterson and Seligman [1] study that their classification of the character strengths and virtues identified would be modified in the light of future further studies and this is indeed the case, with subsequent empirical research finding that character strengths may be better categorized into models with between five [24,25], four [26], and three [27] factors. Duan et al. [12] extended these studies in Mainland China by examining the factor structure and functional equivalence of a Simplified Chinese version of the VIA-IS (the six-virtue factor model by Peterson and Seligman [1]). However, they determined that the 24 strengths could be grouped under three virtues (interpersonal, vitality, and conscientiousness) in a Chinese context. Culturally appropriate measurements should be adopted, so this study used a three-factor CVQ-96 to assess Chinese virtues in a Hong Kong context.

### 2.3. Virtues and Resilience

Resilience is a multi-faceted [28] and unstable construct [29], which also has many interpretations and definitions, such as being able to bounce back or recover from stress [30], to remain in good form during adversity, to respond positively to challenging or adverse situations [31], and to use positive adaptation to reach beneficial outcomes of engagement, commitment, agency, enthusiasm, and well-being [32]. The concept of resilience has also been employed as an important attribute in education and personal development and has been related to positive well-being indicators [33,34,35].

It is stated that “strengths and virtues determine how an individual copes with adversity” [36] (p. 65). An examination of the literature reveals that although the important role of character strengths and virtues has been documented for students, prior studies have failed to examine the relationships between virtues and student resilience in the Hong Kong context. Previous studies have shown that virtues such as courage and temperance positively predicted psychological resilience [37] and that character strengths could predict resilience and other related factors [38]. Personality strengths and virtues can also serve as cushions against life stresses and challenges, which could be conducive to resilient outcomes [39] in different contexts. This study, therefore, assessed the relationships between students’ perceptions of virtues and resilience in the Chinese context.

### 2.4. The Role of Gender and School Level on Virtue and Resilience

Although the positive relationships between virtue and resilience have been examined in different contexts, how demographic factors, such as gender or school grade level, influence the ways in which protective factors (i.e., virtue or character strength) are associated with resilience [8] has received less attention. The following section examines these two demographic factors: gender and school stage.

Various studies have identified the relationships among gender, virtue, and resilience. For instance, male students tend to have slightly higher mean scores for hope than female students [40]. In contrast, Lee and Huang [38] found that female students tend to reveal greater strengths (higher scores) than male students. Female students also tend to attain higher scores for resilience [41,42].

From the perspective of grade level, mean scores for hope showed a declining trend from primary to junior secondary and senior secondary levels [40]. In contrast, Shoshani and Shwartz [43] found that differences were detected between children between 7 and 8–9 years old (junior primary) and those between 9 and 12 years old (around senior primary), which might be attributed to cognitive and emotional maturity, as well as the possible increase of interpersonal relationships with age. Furthermore, younger students in Year 3 had notably higher resilience scores than Years 5 and 7 students for communication, empathy, help-seeking; school support; prosocial peers; meaningful participation in school activities; and autonomy experiences [44].

As mentioned above, because both gender and school grade level affect the results in different contexts, this study examined their role in Chinese virtues and resilience in a Hong Kong context to answer the following three research questions.

RQ1: To what extent do Chinese virtues affect student resilience?
RQ2: What is the role of gender in Chinese virtues and student resilience?
RQ3: What is the role of school grade level in Chinese virtues and student resilience?

## 3. Method

### 3.1. Participants and Procedure

This study was part of a larger study investigating students’ perceptions of values and well-being in Hong Kong. The study adopted a convenience sampling method. Schools which agreed to participate have first be briefed with the project aims and requirements, then the school principals have returned their consent for participation. Once consents were sought, at least one class in each grade was selected to participate in the study. Students were accessed through their class teachers to fill out and return the questionnaire in classroom. An instruction guideline had been provided to school teachers, to ensure they guide the students to complete the survey with the same protocol. Every student who was aged below 18 was required to return a parental consent form before they completed the questionnaire; students without parental consent were not allowed to be one of the participants.

The total sample consisted of 2468 students from 40 primary and 30 secondary schools in Hong Kong, China, with 1143 males (46.6%) and 1309 females (53.4%). Among them, 660 (26.7%) students were from primary schools, including 247 students from Grade 4, 241 students from Grade 5, and 172 students from Grade 6; 1808 (73.3%) were from secondary schools, consisting of 324 students from Grade 7, 404 students from Grade 8, 286 students from Grade 9, 487 students from Grade 10, and 307 students from Grade 11. Grade 12 (Secondary 6) students were not included, as they were studying for public examinations. All the students were well informed about the purpose of the research and participated in this project voluntarily.

### 3.2. Measures

The 96-item CVQ-96 [12,45] consists of three factors or categories of virtues, namely relationship/interpersonal (32 items), vitality (40 items), and conscientiousness/caution (24 items). Students rated the CVQ-96 on a five-point Likert scale, ranging from 1 (very much unlike me) to 5 (very much like me). The Cronbach’s alpha coefficients of the three subscales in this study were 0.951, 0.950, and 0.901 for relationship, vitality, and conscientiousness, respectively. The brief resilience scale (BRS), developed by Fung [46], is a simplified resilience scale consisting of six items. Students were requested to rate to what extent they agreed or disagreed with each statement on a five-point Likert scale, ranging from 1 (strongly disagree) to 5 (strongly agree). The Cronbach’s alpha coefficient for the scale was 0.67.

### 3.3. Data Analysis

Data analysis was performed using SPSS 27 and Mplus 8. The descriptive statistics, including mean (M) and standard deviation (SD), were calculated using SPSS 27. We also conducted confirmatory factor analysis (CFA) on the participants’ responses to the CVQ-96 and BRS via Mplus 8. Structural equation modeling (SEM) was employed to estimate the relationship between these two variables. The following indices were used to determine acceptable model fit: comparative fit index (CFI > 0.90), the Tucker–Lewis index (TLI > 0.90) [47,48,49], and the root mean square error of approximation (RMSEA < 0.08) [50]. The bootstrap method (bootstrap = 5000) was used to calculate the estimates of the SEM with a 95% confidence interval. If the confidence interval included 0, the coefficient was deemed insignificant.

## 4. Results

### 4.1. Descriptive Statistics

In the CVQ-96 sub-scales, students scored highest on relationship (*M* = 3.65, *SD* = 0.56), followed by vitality (*M* = 3.39, *SD* = 0.58), and scored the lowest on conscientiousness (*M* = 3.27, *SD* = 0.55) (Table 1). Fairness ranked the highest (*M* = 3.81, *SD* = 0.66) and gratitude (*M* = 3.44, *SD* = 0.74) ranked the lowest for relationships. Curiosity ranked the highest (*M* = 3.52, *SD* = 0.77), while belief (*M* = 3.20, *SD* = 0.84) ranked the lowest for vitality. Prudence ranked the highest (*M* = 3.37, *SD* = 0.71), while learning (*M* = 3.10, *SD* = 0.85) ranked the lowest for conscientiousness. Female students (*M* = 3.70, *SD* = 0.52) scored higher on the relationship sub-scale than male students (*M =* 3.59, *SD* = 0.60), and the differences were statistically significant (*t* = −4.480, *p* < 0.001). However, no statistically significant differences were found for vitality and conscientiousness with regard to gender. The values for each dimension of the CVQ-96 among primary school students (*M*_relationship_ = 3.74, *SD*_relationship_ = 0.66; *M*_vitality_ = 3.54, *SD*_vitality_ = 0.65; *M*_conscientiousness_ = 3.36, *SD*_conscientiousness_ = 0.63) were higher than those among secondary school students (*M*_relationship_ = 3.62, *SD*_relationship_ = 0.52; *M*_vitality_ = 3.33, *SD*_vitality_ = 0.54; *M*_conscientiousness_ = 3.23, *SD*_conscientiousness_ = 0.51), and the differences between school type were also significant (*t*_relationship_ = 4.20, *p* < 0.001; *t*_vitality_ = 7.40, *p* < 0.001; *t*_conscientiousness_ = 4.74, *p* < 0.001).

On the BRS, participants reported adequate resilience (*M* = 3.19, *SD* = 0.57). Male students (*M*_male_ = 3.22, *SD*_male_ = 0.59) scored higher than female students (*M*_female_ = 3.16, *SD*_female_ = 0.56) with statistical significance (*t* = 2.26, *p* < 0.05). The scores from primary schools (*M*_primary_ = 3.27, *SD*_primary_ = 0.64) were higher than those from secondary schools *M*_secondary_ = 3.16, *SD*_secondary_ = 0.54), and the differences were also statistically significant (*t* = 4.09, *p* < 0.001).

To further understand the impact of the school level, the role of grade level was also examined. The outcome showed students from Grade 4 scored the highest on all three sub-scales (i.e., relationship, vitality, and conscientiousness) in the CVQ-96, while students from Grade 9 scored the lowest on relationship, and students from Grade 11 scored the lowest on both vitality and conscientiousness (Table 2). In general, the mean values for the CVQ-96 scores indicated a decreasing trend by grade, which means that students from higher grades reported lower scores.

One-way ANOVA was used to test the differences in school grade levels on the three subscales of the CVQ-96. The results revealed statistical differences in relationship, vitality, and conscientiousness between at least two groups (relationship: *F*_(7, 2460)_ = 6.92, *p* = < 0.001; vitality: *F*_(7, 2460)_ = 17.80, *p* = < 0.001; conscientiousness: *F*_(7, 2460)_ = 11.29, *p* < 0.001). For relationship, the results of Dunnett T3’s post hoc test indicated that the mean values were significantly different between Grade 4 and other grades except for Grade 6. As for vitality, the mean values were significantly different between Grade 4 and other grades. The differences in Grade 5, Grade 7, and Grade 11 were also significant. For conscientiousness, the mean values were significantly different between Grade 4 and other grades, as well as between Grade 11 and other grades, except for Grade 6 and Grade 9.

### 4.2. CVQ-96 Measurement Model

**Figure 1:**
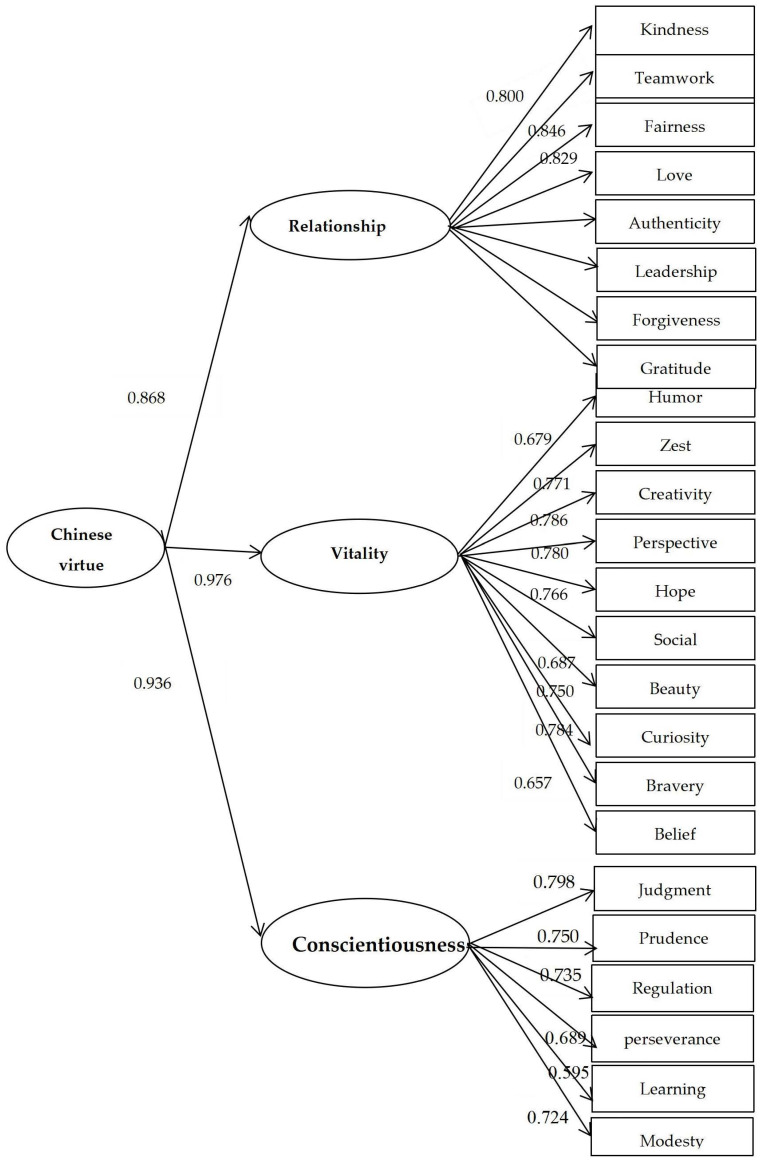
Measurement Model for Chinese Virtues.

Poor model fit was identified when we conducted CFA for the original three-factor structure of the CVQ-96 (CFI = 0.785, TLI = 0.781, RMSEA = 0.058). Three alternative models were then derived using different methods, and the details of the model fit indexes are summarized in Table 3. Because the scale contains a large number of items, it is difficult to achieve good model fit, so item parceling was used to stabilize parameter estimates and to improve model fit [51,52]. Based on the item parceling method, the revised model was finally composed of second-order factors (Figure 1), which yielded an acceptable model fit (CFI = 0.905, TLI = 0.893, and RMSEA = 0.083 with 90% CI [0.081, 0.085]). All the factor loadings in the proposed second-order model were significant and acceptable.

### 4.3. BRS Measurement Model

**Figure 2:**
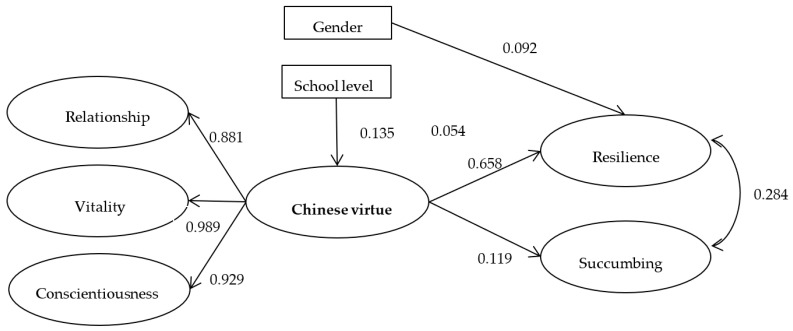
SEM Model of Chinese Virtues and Resilience.

The original factor structure of the BRS did not show a good model fit in CFA. Therefore, a model with two factors, divided into positive resilience and succumbing based on Fung [46], was used to verify the factor structure (see the right side of Figure 2). The outcome was satisfactory (CFI = 0.987, TLI = 0.975, RMSEA = 0.061, 90% CI [0.050, 0.074]).

### 4.4. SEM Results

#### 4.4.1. Common Method Variance

The widely used Harman’s single-factor test was applied to address the issue of common method variance [53]. One factor explained 30.11% of the total variance, which was less than the recommended threshold of 0.5 [54], so it appears that common method bias was not a significant problem in this study.

#### 4.4.2. SEM Results

This study examined the relationship between the CVQ-96 and BRS. The model fit was acceptable (CFI = 0.905, TLI = 0.896, RMSEA = 0.044 with 90% CI [0.042, 0.045]) (Figure 2). The results indicated that the items measured by the CVQ-96 positively affected both resilience (*β* = 0.658, 95% CI [0.614, 0.699]) and succumbing (*β* = 0.119, 95% CI [0.052, 0.185]), as measured by the BRS (Table 4).

The outcome also indicated that the participants’ gender did not show statistically significant differences for the CVQ-96, while gender (*β* = 0.092, 95% CI [0.045, 0.139]) significantly affected positive resilience. Boys appeared to be more resilient than girls. No statistically significant differences were found for the negative resilience factor, succumbing, by gender.

The difference in school grade level significantly affected scores measuring Chinese virtues (*β* = 0.135, 95% CI [0.089, 0.180]) and resilience (*β* = 0.054, 95% CI [0.011, 0.095]). Students from primary school scored higher on both Chinese virtues and positive resilience. No statistically significant differences in succumbing were found in terms of the school grade level.

## 5. Discussion

The current study identified the relationships among Chinese virtues, student resilience, gender, and school grade level using a sample of primary and secondary school students from Hong Kong, China. The rest of this section is organized based on the three research questions.

The impact of Chinese virtues on student resilience was confirmed, addressing the first research question. In the SEM model, the results established the relationship between Chinese virtues and student resilience and indicated that Chinese virtues positively affected students’ positive resilience, which is consistent with previous studies [37,38]. Surprisingly, this study identified a direct positive effect of Chinese virtues on students’ succumbing; this may not be consistent with previous studies [55] that found that character strengths (i.e., the different aspects of virtues), as a multidimensional construct, significantly and jointly predicted resilience. The opposite results in this study might be partly due to Chinese Confucian culture. For instance, in *The Analects*, Kongzi (Confucius), it was determined that *ren* (kindness) is a key component of harmony and good relationships [56], which is also consistent with the previous finding that kindness (i.e., one item from the CVQ-96) decreased resilience [7]. However, this potentially contradictory relationship between Chinese virtues and students’ succumbing requires further analysis in future research.

The role of gender in the relationship between Chinese virtues and student resilience was also demonstrated, addressing the second research question. A positive relationship was found between gender and students’ positive resilience in the SEM model. Male students scored higher than female students for resilience, which matches that of prior studies [57,58], which also found that resilience was significantly higher in males. The reason for this finding could be that girls are generally more emotional than boys so they could be affected more deeply after experiencing adversity or challenges. However, these results are inconsistent with numerous studies in different contexts [42,44], so the contradictory effects of gender in schooling need further investigation.

Surprisingly, there were no relationships between gender and students’ Chinese virtues, which is inconsistent with numerous studies [40,41]. However, when examined more closely, gender had a significant impact on the relationship sub-dimension of the CVQ-96, although gender did not show a significant influence on Chinese virtues as a whole. Consistent with this result, Zhang et al. [45] identified no significant gender differences in scores for vitality and conscientiousness (two sub-dimensions of the CVQ-96) among undergraduate students in Mainland China. This result may be extended to the primary and secondary school levels. Female students also showed higher scores than males in the relationship sub-dimension, and the differences were statistically significant, which is consistent with previous studies that found girls attained higher scores than boys in the character strengths of kindness [41], perhaps because, from an evolutionary perspective, female strengths are shown in the areas of kindness and love, which are linked to the traditional female role of the caregiver [59,60].

The role of school grade level on the relationship between Chinese virtues and student resilience was also verified, answering the third research question. According to our results, the direct effects of school level on Chinese virtues were significant, and the values for each dimension of the CVQ-96 were higher among primary school students than among secondary school students. This has been empirically demonstrated in previous studies [40,61] and agrees with the disruption hypothesis for personality development [62]. These results also indicate the direct influence of school level on student resilience, as prior evidence had demonstrated [44]. Interestingly, the CVQ-96 and resilience scores were both higher among primary school students than among secondary school students. A possible influencing factor for this may be that coping strategies are developed in younger primary school children, and resilience may be subject to fluctuation at the onset of adolescence [44]. To some degree, this shows that school influences students’ Chinese virtues and resilience, which seems to be broadly in line with a previous cross-cultural study of the perceptions of resilience-promoting factors among students, which found a relatively strong relationship between teachers, students, and parents in a Chinese sample [63] (p. 15). In another study in Hong Kong, it was found that, despite a small decrease in resilience during adolescence, the strengthening of family processes assists in promoting resilience in this age group [64]. In addition, this study was partly based on the assumption that Chinese virtues or individual strengths, as psychological *suzhi* (psychological quality), might serve as protective factors that could counteract, either positively or negatively, the adverse outcomes related to risk [65] (pp. 357, 366). However, some research findings suggest cautionary interpretations when applying resilience theory to a Chinese context [65] (p. 368). Thus, future research should more deeply consider the relationship between Chinese virtues and resilience during early adolescence.

In the SEM model, school grade level showed a significant impact on Chinese virtues, which in turn affected resilience. This is in line with the findings that grade differences affect the association with student virtues [40] and character strengths could predict resilience [38]. However, few studies have investigated the role of school level in the relationship between Chinese virtues and student resilience, so this study makes a valuable contribution to our understanding of the role of Chinese virtues and the related influential factors and outcomes.

## 6. Theoretical Implications

This study had four major implications. First, the potential linkage between virtues and resilience contributed to the conceptual understanding that enhancing students’ resilience could be achieved through nurturing virtues and the related character strengths. This emphasized the importance of students’ virtues and resilience. Second, this study illustrated the role of gender and school level on the relationship between Chinese virtues and student resilience. Future studies could investigate the overall effect of the interaction of school level and gender on virtues and resilience. Third, the positive relationship between virtues and resilience supported further character education in schools. Based on successful interventions (e.g., strengths-based resilience programs by Rashid et al. [66]; the Bounce Back Well-being and Resilience program by McGrath and Noble [67]; and the “You Can Do it!” program by Noble and McGrath [68]), this study provided information for policymakers and schools to target policies, programs, and interventions to improve the virtues and resilience of their students.

Fourth, there was certain compatibility or overlap between the character and virtue approach [69] from the Western literature and the policy texts on Chinese moral education for primary and secondary schools under the influence of Confucian heritage. Some scholars have remarked that different Chinese philosophies, such as Confucianism, Buddhism, and Taoism, tend to endorse the acceptance of adversity, and its processes tend to be related to relationships with others and with nature, instead of being individualistic [70]. Future studies could further examine the Hong Kong context, where there are the possibilities and prospects of learning from Chinese moral and life education [71] and positive character education from the West [72].

## 7. Practical Implications

The Ministry of Education, educators, and schools could work together to develop programs or curricula related to resilience and character education to help students learn how to respond to and cope with potential harms and adversity, thus enhancing students’ resilience and character strength [73,74,75]. For example, storytelling and drama could be incorporated into character or resilience education [76,77,78]. Students could absorb values by listening to or retelling stories. Storytelling can also develop students’ cognitive, affective, and psychomotor domains, and drama could provide a meaningful, multisensory, hands-on learning experience [78] directly related to character or resilience education.

For teachers, hostile climates in the classroom should be avoided, as this might cause psychological, social, and epistemic harm to students instead of developing the necessary skills for dealing properly with challenging environments [75,79]. For parents, their warmth, monitoring, and school involvement benefit the development of their children’s character [80]. Teachers and parents should work together with the school to create situations for students to experience failure and success as well as teach them different strategies to protect themselves, such as “battle on through”, “believe in yourself”, “just don’t listen”, and “keep your head down” [75].

## 8. Limitations and Future Research

Despite this study’s contributions, this investigation had several limitations. Methodologically, the study adopted a convenience sampling method; because the sample was not randomly selected, caution should be used when generalizing the results to other fields. The study depended on cross-sectional and self-reported questionnaire data from students. Causal relationships among measures could not be inferred. Future studies could include cognitive interviews or use longitudinal data together with other measures to ascertain the relationships between virtues and resilience in the Chinese context. This study only examined the general effect of Chinese virtues on students’ resilience; thus, future studies could consider the influence of virtues from the different sub-dimensions, such as interpersonal, vitality, and conscientiousness.

## 9. Conclusions

This paper assessed the applicability of the CVQ-96 [9] and the BRS [27] on a sample of primary and secondary school students in Hong Kong and explored the possible relationships between the Chinese virtues and resilience as affected by school grade level and gender differences. The current study examined the relationships among Chinese virtues, student resilience, gender, and school grade level. Boys were more resilient than girls. Students from primary school were more resilient and scored higher on the CVQ-96 than students from secondary school. With the increment of Chinese virtues, students’ positive resilience and succumbing also increased. The results could be applied directly to guide the development of resilience and gender education.

## Figures and Tables

**Table 1:** Descriptive Statistics for the Chinese Virtues Questionnaire and Brief Resilience Scale.

Chinese Virtue (and Its Sub-Scales)	Character Strength Items	MEAN	SD	Female	Male	Secondary	Primary	Model Fit Second Order
Relationship/Interpersonal	Kindness	3.56	0.66	3.63 (0.63) ***	3.48 (0.70)	3.54 (0.62) **	3.63 (0.77)	CFI = 0.905 TLI = 0.897 RMSEA = 0.079 90% CI: 0.077–0.080
	Teamwork	3.72	0.69	3.78 (0.65) ***	3.66 (0.73)	3.69 (0.65) ***	3.81 (0.78)	
	Fairness	3.81	0.66	3.87 (0.62) ***	3.76 (0.7)	3.79 (0.62) **	3.89 (0.76)	
	Love	3.65	0.72	3.73 (0.70) ***	3.55 (0.74)	3.62 (0.69) **	3.73 (0.81)	
	Authenticity	3.63	0.64	3.67 (0.60) ***	3.58 (0.69)	3.61 (0.61)	3.66 (0.72)	
	Leadership	3.70	0.68	3.75 (0.63) **	3.66 (0.72)	3.67 (0.63) ***	3.80 (0.78)	
	Forgiveness	3.70	0.68	3.72 (0.65)	3.67 (0.71)	3.66 (0.63) ***	3.81 (0.79)	
	Gratitude	3.44	0.74	3.46 (0.72)	3.42 (0.76)	3.39 (0.71) ***	3.58 (0.81)	
	Overall	3.65	0.56	3.70 (0.52) ***	3.60 (0.60)	3.62 (0.52) ***	3.74 (0.66)	
Vitality	Humor	3.42	0.80	3.37 (0.77) **	3.48 (0.82)	3.39 (0.76) ***	3.52 (0.88)	CFI = 0.843 TLI = 0.833 RMSEA = 0.082 90% CI 0.080–0.083
	Curiosity	3.52	0.77	3.51 (0.76)	3.53 (0.77)	3.42 (0.72) ***	3.77 (0.82)	
	Zest	3.38	0.76	3.34 (0.75) **	3.44 (0.77)	3.28 (0.71) ***	3.66 (0.83)	
	Creativity	3.41	0.73	3.37 (0.71) **	3.45 (0.75)	3.36 (0.69) ***	3.52 (0.82)	
	Perspective	3.30	0.69	3.32 (0.66)	3.28 (0.72)	3.28 (0.65) *	3.35 (0.79)	
	Hope	3.37	0.75	3.34 (0.75) *	3.42 (0.76)	3.31 (0.72) ***	3.55 (0.82)	
	Social	3.45	0.68	3.49 (0.65) **	3.42 (0.7)	3.44 (0.64)	3.48 (0.77)	
	Beauty	3.43	0.74	3.50 (0.71) ***	3.35 (0.76)	3.39 (0.70) ***	3.54 (0.83)	
	Bravery	3.38	0.70	3.34 (0.68) **	3.43 (0.72)	3.33 (0.66) ***	3.52 (0.78)	
	Belief	3.20	0.84	3.17 (0.82)	3.23 (0.86)	3.10 (0.81) ***	3.47 (0.85)	
	Overall	3.39	0.58	3.37 (0.55)	3.40 (0.61)	3.33 (0.54) ***	3.54 (0.65)	
Conscientiousness/Caution	Judgment	3.35	0.67	3.37 (0.62)	3.32 (0.73)	3.36 (0.63)	3.31 (0.79)	CFI = 0.881 TLI = 0.866 RMSEA = 0.084 90% CI 0.082–0.086
	Prudence	3.37	0.71	3.39 (0.68)	3.35 (0.75)	3.37 (0.67)	3.39 (0.81)	
	Regulation	3.17	0.69	3.13 (0.69) **	3.22 (0.70)	3.11 (0.66) ***	3.32 (0.76)	
	Perseverance	3.35	0.66	3.32 (0.64) *	3.38 (0.69)	3.31 (0.64) ***	3.45 (0.73)	
	Learning	3.10	0.85	3.10 (0.84)	3.09 (0.85)	3.01 (0.81) ***	3.32 (0.91)	
	Modesty	3.27	0.67	3.28 (0.63)	3.26 (0.72)	3.23 (0.62) ***	3.38 (0.78)	
	Overall	3.27	0.55	3.26 (0.50)	3.27 (0.59)	3.23 (0.51) ***	3.36 (0.63)	
Resilience		3.19	0.57	3.16 (0.56) *	3.22 (0.59)	3.16 (0.54) ***	3.27 (0.64)	CFI = 0.612 TLI = 0.354 RMSEA = 0.314 90% CI 0.303–0.325

Note: * *p* < 0.05; ** *p* < 0.01; *** *p* < 0.001.

**Table 2:** Means and Standardization for Chinese Virtues Questionnaire by Grade.

Grade	N	Relationship	Vitality	Conscientiousness
1 Primary 4	247	3.85 (0.69)	3.67 (0.69)	3.51 (0.67)
2 Primary 5	241	3.64 (0.65)	3.47 (0.60)	3.31 (0.59)
3 Primary 6	172	3.72 (0.59)	3.43 (0.60)	3.23 (0.58)
4 Secondary 1	324	3.65 (0.58)	3.43 (0.58)	3.30 (0.58)
5 Secondary 2	404	3.67 (0.5)	3.39 (0.53)	3.27 (0.49)
6 Secondary 3	286	3.58 (0.54)	3.33 (0.54)	3.20 (0.53)
7 Secondary 4	487	3.59 (0.51)	3.31 (0.51)	3.24 (0.48)
8 Secondary 5	307	3.59 (0.48)	3.19 (0.50)	3.13 (0.45)

**Table 3:** Model Fit Indices for the Chinese Virtues Questionnaire and Brief Resilience Scale.

	Chinese Virtues Questionnaire				Brief Resilience Scale	
	Model 1 First Order	Model 2 Second Order	Model 3 Third Order	Model 4	1-Factor	2-Factor
CFI	0.785	0.820	0.821	0.905	0.61	0.987
TLI	0.781	0.815	0.816	0.893	0.35	0.975
RMSEA	0.058 (0.057–0.058)	0.053 (0.053–0.054)	0.053 (0.052–0.054)	0.083 (0.081–0.085)	0.314 (0.303–0.325)	0.061 (0.050–0.074)

**Table 4:** Standardized Coefficients and 95% Confidence Intervals for SEM model of Resilience and Chinese Virtues (bootstrap = 5000).

	Resilience 1		Resilience 2		Chinese Virtues	
Predictor	β	95% CI	β	95% CI	β	95% CI
School level	0.054	[0.011–0.095]	0.022	[−0.031–0.073]	0.135	[0.089–0.180]
Gender	0.092	[0.045–0.139]	−0.021	[−0.068–0.026]		
Chinese virtue	0.658	[0.614–0.699]	0.119	[0.052–0.185]		

## Data Availability

The data is unavailable due to privacy or ethical restrictions.
